# Achieving blood preSsure goals sTudy in uncontrolled hypeRtensive pAtients treated with a fixed-dose combination of ramipriL/hydrochlorothiazide: the ASTRAL study

**DOI:** 10.5830/CVJA-2010-086

**Published:** 2011-04

**Authors:** IG Okpechi, BL Rayner, HS Schoeman, B Longo-Mbenza, DA Oke, S Kingue, JL Nkoua

**Affiliations:** Division of Nephrology and Hypertension, Groote Schuur Hospital, University of Cape Town, South Africa; Division of Nephrology and Hypertension, Groote Schuur Hospital, University of Cape Town, South Africa; Clinical Statistics, Doornpoort, Pretoria, South Africa; Faculty of Health Sciences, Walter Sisulu University, Mthatha, Eastern Cape, South Africa and Department of Internal Medicine, University Hospital of Kinshasa, Democratic Republic of Congo; Division of Medicine, Lagos University Teaching Hospital, Lagos, Nigeria; Division of Medicine, General Hospital and University of Yaoundé, Cameroon; Division of Cardiology, University Hospital of Brazzaville, Congo

**Keywords:** hypertension, ramipril, black Africans, ACE inhibitors, thiazides

## Abstract

**Background:**

Hypertension is a common cardiovascular disease, affecting adults worldwide and it accounts for up to 30% of all deaths. The need for better control of arterial hypertension justifies observational studies designed to better understand the real-life management of hypertensive patients. The ASTRAL study was primarily designed to evaluate the percentage of hypertensive patients achieving blood pressure goals after eight weeks of treatment with a fixed-dose combination of ramipril/hydrochlorothiazide (HCTZ).

**Methods:**

The study was a multi-centre, non-comparative, open-label, observational study conducted in 36 centres in five sub-Saharan African countries, namely Cameroon, Congo Brazzaville, Democratic Republic of Congo (DRC), Madagascar and Nigeria. Four hundred and forty-nine men and women 18 years of age or older with hypertension not controlled by an ACE inhibitor, a diuretic or any other monotherapy or anti-hypertensive combination not containing a diuretic in a fixed dose were considered eligible for inclusion in this eight-week study. The study consisted of three visits, visit one (V1) at baseline, visit two (V2) after four weeks and visit three (V3) after eight weeks.

**Results:**

The mean age of the patients was 54.7 ± 11.7 years (20–90 years) and most were categorised by the WHO criteria as either overweight or obese (71.6%). After four and eight weeks of treatment with the study drug, systolic and diastolic blood pressures significantly changed from baseline: –24.7/–14.2 mmHg (*p* < 0.001) and –31.7/–17.9 mmHg (*p* < 0.001), respectively. There were 60.2% of the non-diabetics on prior monotherapy who, at eight weeks, fulfilled the primary blood pressure goal for SBP and DBP, versus 26.5% of the diabetic patients, also on monotherapy. Few adverse events were reported, with facial oedema and dry cough recurring twice in two patients.

**Conclusion:**

Fixed-dose combination of ramipril/HCTZ is therefore effective, tolerable and has a good safety profile for blood pressure control in black Africans.

## Abstract

Hypertension is the most prevalent cardiovascular (CV) disease in adults worldwide and is a major risk factor for both cardiovascular and cerebrovascular morbidity and mortality.^1^ Systemic arterial hypertension is globally estimated to affect 30% of adults^2^,^3^ and to account for up to 30% of all deaths.^1^ Although the results of several cross-sectional and cohort epidemiological studies show that the prevalence of hypertension varies significantly, 4–9 the prevalence in sub-Saharan Africa varies between 12 and 29%, depending on the country.^10^ In developed countries, fewer than 27% of patients with arterial hypertension have controlled blood pressure (BP),^3^ whereas in developing countries this number is less than 10%.^11^ The relationship between BP and CV risk is continuous, such that every 20-mmHg rise in systolic blood pressure (SBP) or 10-mmHg rise in diastolic blood pressure (DBP) doubles the risk of cardiovascular disease (CVD).^12^ Therapy with anti-hypertensive drugs have shown a 35 to 40% reduction in stroke, a 20 to 25% reduction in myocardial infarction, a more than 50% reduction in heart failure, and reductions in CVD-related death rates.^3^

Age, ethnicity, obesity, smoking, excessive consumption of alcohol and physical inactivity are among the risk factors identified to be associated with hypertension. The JNC-7 and recent European guidelines^3^,^11^ have recommended BP goals at the following levels: < 140/90 mmHg in non-diabetic hypertensive patients, and < 130/80 mmHg in diabetic hypertensive patients. Both the JNC-7 and the European guidelines have also recommended BP treatment with at least two agents or using a combination of diuretic and another antihypertensive agent that has a different mode of action for patients presenting with BP values ≥ 20/10 mmHg above the BP goals.^3^,^11^ Additionally, fixed-drug combinations offer several advantages over the separate prescription of individual drugs.^11^ The need for better control of arterial hypertension justifies observational studies designed to better understand the real-life management of hypertensive patients, especially in Africa, where few hypertension studies have been conducted.

The primary outcome of the ASTRAL study was to evaluate the percentage of hypertensive patients achieving BP goals after eight weeks of treatment with a fixed-dose combination of ramipril/HCTZ, hence to generate data on the effectiveness of the fixed-dose combination.

Ethics committee approval to undertake the survey was obtained from hospitals in each country in accordance with national and local regulations. Written, signed consent was obtained from each of the patients included. The study was conducted in accordance with the Helsinki II Declaration.

## Methods

**Fig. 1.**
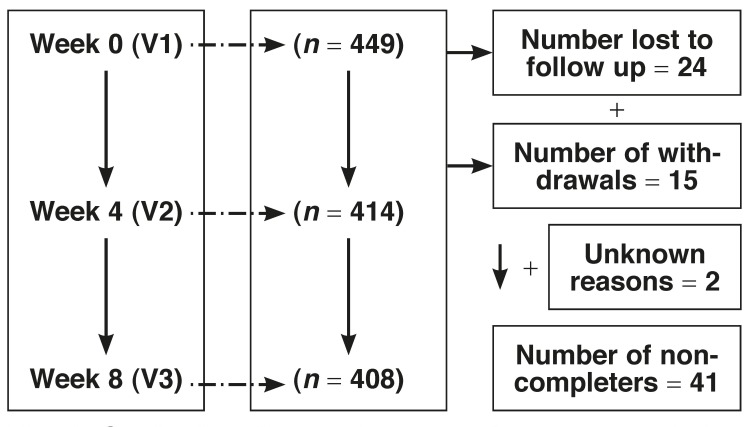
Study time line and reasons for non-completion of study.

The study was a multi-centre, non-comparative, open-label, observational study conducted across 36 centres in five sub-Saharan African countries (Cameroon, Congo Brazzaville, DRC, Madagascar and Nigeria). Men and women 18 years of age or older with arterial hypertension uncontrolled by an ACE inhibitor, a diuretic, or any other monotherapy or anti-hypertensive combination not containing a diuretic in a fixed dose were considered eligible for inclusion in the study. Exclusion criteria were: known hypersensitivity to any component of the study drug (Tritazide®), history of angio-oedema, patients with severe renal or hepatic dysfunction, severe gout, secondary hypertension, and all pregnant or breastfeeding women. Although the study was initially planned to include 460 consecutive hypertensive patients from the 36 participating centres, only 449 patients could be recruited and included to receive the study drug; 408 (90.9%) of these patients completed the study. The reasons for non-completion of the study are summarised in Fig. 1.

Three visits were scheduled during the study. At visit one (week 0), demographic data, history of co-morbidities, duration of hypertension, blood pressures at first diagnosis of hypertension and previous anti-hypertensive treatment given, concomitant medications, and CV risk factors were recorded. SBP and DBP were measured and recorded in the seated position after five minutes of rest in the medical office, using a validated and calibrated electronic sphygmomanometer (OMRON M7, OMRON, Tokyo, Japan) with an appropriate cuff size. A blood pressure target was established, recommended lifestyle modifications were given to the patient and the ramipril/HCTZ daily dose was prescribed, based exclusively on the decision of the treating physician and in accordance with the prescribing information in the summary of the product characteristics or package insert. Body weight was recorded in kilograms and height was measured in metres to determine the body mass index (BMI) which was recorded as overweight if ≥ 25 kg/m^2^ and obese if ≥ 30 kg/m^2^, according to the WHO guidelines.^13^

At the follow-up visits at weeks four and eight (visits two and three, respectively), BP was measured, adverse events were assessed and treatment compliance rating was evaluated by taking into account the unused number of tablets returned by the patient on the follow-up visit and expressed as good (< four of 28 tablets returned), average (five to nine of 28 tablets returned) or poor (≥ 10 of 28 tablets returned). The necessity for titration to a higher dose of the study drug was evaluated in response to BP goal.

The primary outcome of the ASTRAL study was to evaluate the percentage of hypertensive patients achieving BP goals after eight weeks of treatment with a fixed-dose combination of ramipril/HCTZ. Blood pressure control was defined as SBP < 140 mmHg and/or DBP < 90 mmHg in non-diabetics and SBP < 130 mmHg and/or DBP < 80 mmHg in diabetic patients. The secondary objectives included the assessment of variations in SBP and DBP values between visits, compliance with treatment, tolerance of the treatment and the factors influencing BP control.

## Statistical analysis

The statistical analysis was performed by ClinStat CC, Pretoria, South Africa. All analyses were carried out on SAS (release 9.1.3). Descriptive statistical analyses were performed. Qualitative variables were expressed as frequency counts and percentages. Quantitative variables were summarised by mean values, standard deviations, minimum and maximum values. Within-subject comparisons (changes in blood pressures at weeks four and eight relative to baseline) were tested for significance by the paired *t*-test.

A logistic regression analysis was performed with goal achievement as a dependent variable and the following predictor variables: age, gender, history of diabetes, alcohol consumption, renal dysfunction, diabetic nephropathy, microalbuminuria, cerebrovascular accident and heart failure, BMI, concomitant treatment with NSAIDs, daily dose of study drug and BP at inclusion. In the logistic regression analysis, the following predictor variables were numeric: age, systolic blood pressure, diastolic blood pressure and daily doses of study drugs. All the other predictor variables were categorical. Adverse events were listed, indicating intensity, relationship with the study drug, discontinuation in the study, corrective treatment, outcome and seriousness. A *p*-value < 0.05 was considered as significant.

## Results

The baseline demographic and clinical features, co-morbidities and associated CV risk factors of all the patients and for patients from the five participating countries are shown in Tables 1 and 2. Four hundred and forty-nine hypertensive patients took part in the study, with a mean age of 54.7 ± 11.7 years (range 20–90 years) and with 11.1% of all the participants being ≤ 40 years old. The mean duration of hypertension was 6.2 ± 6.5 years and there was a slight female preponderance in the study population (male = 44.1%). The mean BMI was 28.1 ± 5.3 kg/m^2^ and most of the patients were categorised as either overweight or obese (72%).

**Table 1.** The Baseline Demographic And Clinical Characteristics Of The Patients

*Characteristic*	*All (n = 449)*	*Cameroon (n = 100)*	*Congo (n = 87)*	*DRC (n = 62)*	*Madagascar (n = 100)*	*Nigeria (n =100)*
Age (years)	54.7 ± 11.7	55.2 ± 11.1	58.3 ± 13.1	54.0 ± 12.3	51.7 ± 10.4	54.2 ± 11.3
Male (%)	198 (44.1)	46 (46.0)	31 (35.6)	27 (43.5)	44 (44.0)	50 (50.0)
BMI (kg/m^2^)	28.1 ± 5.3	29.2 ± 5.3	27.6 ± 5.3	28.6 ± 5.6	25.6 ± 3.9	29.9 ± 5.4
Alcohol users (%)	87 (19.4)	32 (32.0)	19 (21.8)	10 (16.1)	8 (8.0)	18 (18.0)
Duration of hypertension (years)	6.2 ± 6.5	4.4 ± 5.2	7.2 ± 7.8	5.9 ± 4.9	7.1 ± 5.6	6.6 ± 7.9
Number of anti-hypertensive agents in use:						
0	40 (8.9)	10 (10.0)	–	5 (8.1)	15 (15.0)	10 (10.0)
1	278 (61.9)	50 (50.0)	78 (89.7)	35 (56.4)	72 (72.0)	43 (43.0)
2	99 (22.0)	29 (29.0)	8 (9.2)	18 (29.0)	10 (10.0)	34 (34.0)
3	28 (6.2)	11 (11.0)	1 (1.1)	2 (3.2)	3 (3.0)	11 (11.0)
4	3 (0.7)	–	–	2 (3.2)	–	1 (4.0)
Not sure	1 (0.2)	–	–	–	–	1 (4.0)
SBP (mmHg)	168.9 ± 19.2	178.6 ± 19.9	180.7 ± 24.9	173.7 ± 20.7	174.2 ± 20.4	166.3 ± 22.1
DBP (mmHg)	102.6 ± 12.3	105.0 ± 12.1	103.5 ± 13.0	101.7 ± 12.3	105.0 ± 11.5	101.3 ± 13.0
No using ≤ 1 anti-hypertensive agents (%)	318 (70.8)	60 (60.0)	78 (89.7)	40 (64.5)	87 (87.0)	53 (53.0)

DRC: Democratic Republic of Congo, BMI: body mass index, SBP: systolic BP, DBP: diastolic BP. Values are given as mean ± SD or as number of cases (%).

**Table 2.** Percentages Of Patients With Co-Morbidities And Specified Cardiovascular Risk Factors

*Co-morbidities or CV risk factors*	*All n (%)*	*Cameroon n (%)*	*Congo n (%)*	*DRC n (%)*	*Madagascar n (%)*	*Nigeria n (%)*
Patients with associated co-morbidities	160 (35.6)	32 (32.0)	29 (33.3)	50 (80.7)	14 (14.0)	35 (35.0)
Cerebrovascular accident	31 (6.9)	10 (10.0)	9 (10.3)	3 (4.8)	2 (2.0)	7 (7.0)
Coronary artery disease	6 (1.3)	–	3 (3.4)	2 (3.2)	–	1 (1.0)
Heart failure	29 (6.5)	1 (1.0)	3 (3.4)	19 (30.6)	–	6 (6.0)
Diabetic nephropathy and microalbuminuria	19 (4.2)	5 (5.0)	3 (3.4)	5 (8.0)	–	6 (6.0)
Others	75 (16.7)	16 (16.0)	11 (12.5)	21 (34.1)	12 (12.0)	14 (14.0)
Cardiovascular risk factors						
Overweight/obese	320 (71.6)	63 (63.0)	46 (52.9)	33 (53.2)	54 (54.0)	50 (50.0)
Smokers	21 (4.7)	2 (2.0)	1 (1.1)	2 (3.2)	14 (14.0)	2 (2.0)
Dyslipidaemia	72 (16.0)	9 (9.0)	13 (14.9)	13 (21.0)	24 (24.0)	13 (13.0)
Diabetes	88 (19.6)	7 (7.0)	33 (37.9)	4 (6.4)	24 (24.0)	20 (20.0)
No CV risk factor	77 (17.1)	2 (2.0)	22 (25.3)	17 (27.4)	24 (24.0)	12 (12.0)

DRC: Democratic Republic of Congo.

Patients with a history of diabetes made up 19.6% of the study population and 2.4% were unaware of their diabetes status. There was a high number of patients with associated hypertension-related end-organ damage or co-morbidities (35.6%), such as previous cerebrovascular accidents, coronary artery disease and heart failure, accounting for 6.9, 1.3 and 6.5%, respectively. At the start of the study, only 17.1% of the patients had no known CV risk factor. No statistically significant differences were observed between the different countries in the baseline characteristics of the patients.

At baseline, the mean SBP and DBP were 168.9 ± 19.2 and 102.6 ± 12.3 mmHg, respectively and most of the patients (70.8%) had been on only one or no anti-hypertensive agent before the study. Also, most of the patients (99.1% at V1, 91.5% at V2 and 88.7% at V3) started and remained on a standard dose (5/25 mg) or half-standard dose (2.5/12.5 mg) of the fixeddose combination ramipril/HCTZ for the duration of the study (Table 3).

**Table 3.** Treatment Schedule Of Tritazide, Mean BP Changes From Baseline And Study Drug Compliance.

*Dose of Tritazide (ramipril/HCTZ) (mg)*	*Number (%) of patients*		
	*Week 0 (V 1)*	*Week 4 (V 2)*	*Week 8 (V 3)*
2.5/12.5	173 (38.7)	77 (18.7)	65 (16.0)
5/25	270 (60.4)	299 (72.8)	295 (72.7)
7.5/37.5	–	5 (1.2)	14 (3.4)
10/50	4 (0.9)	30 (7.3)	32 (7.9)
Blood pressures and BP changes from baseline			
SBP (mmHg)	168.9 ± 19.2	143.5 ± 19.7	136.5 ± 15.5
Δ SBP (mmHg)	NA	– 24.7*	–31.7*
DBP (mmHg)	102.6 ± 12.3	88.7 ± 11.9	84.8 ± 9.9
Δ DBP (mmHg)	NA	–14.2*	–17.9*
Study drug compliance	*n* = 449	*n* = 408	*n* = 407
Good	NA	364 (89.2)	377 (92.6)
Medium	NA	34 (8.3)	21 (5.2)
Poor	NA	10 (2.5)	9 (2.2)

HCTZ: hydrochlorothiazide, V1: visit one, V2: visit two, V3: visit three, BP: blood pressure, SBP: systolic BP, DBP: diastolic BP, Δ SBP: change in systolic BP from baseline, Δ DBP: change in diastolic BP from baseline, NA: not applicable; *p < 0.001.

The patterns of BP changes observed in this study were similar across the five participating countries. At V2 and V3, BP significantly changed from baseline: –24.7/–31.7 for SBP mmHg (*p* < 0.001) and –14.2/–17.9 mmHg (*p* < 0.001) for DBP (Table 3). Table 4 shows the mean and changes in SBP and DBP from visit one in patients receiving monotherapy and dual or more therapy before and after commencement of the study. SBP and DBP changes were similar in patients who had been on monotherapy and those who had been on dual or more therapy. There were 60.2% of the non-diabetic patients on prior monotherapy who fulfilled the primary BP goal at V3 for SBP and DBP, versus 26.5% of diabetic patients on monotherapy. Of those patients on prior dual or more anti-hypertensive therapy, 41.5% of the non-diabetics (vs 14.3% of diabetics) met the primary BP goal at visit three (Table 5).

**Table 4.** Comparison Of Blood Pressures Before And After Commencement Of Study Drug In Patients Who Received Monotherapy And Those Who Received Dual Or More Therapy

	*Monotherapy*			*Dual or more therapy*		
	*Visit 1 (n = 267)*	*Visit 2 (n = 247)*	*Visit 3 (n = 238)*	*Visit 1 (n = 142)*	*Visit 2 (n = 131)*	*Visit 3 (n = 132)*
SBP (mmHg)	168.1 ± 19.1	141.9 ± 19.4	134.3 ± 14.8	170.1 ± 20.0	147.1 ± 18.0	139.3 ± 15.8
Δ SBP (mmHg)	NA	–25.3*	–32.9*	NA	–23.0*	–30.4*
DBP (mmHg)	102.0 ± 11.7	88.2 ± 12.1	83.3 ± 9.6	102.4 ± 11.8	89.6 ± 10.7	86.7 ± 9.6
Δ DBP (mmHg)	NA	–14.1*	–18.8*	NA	–13.3*	–16.0*

SBP: systolic BP, DBP: diastolic BP, Δ SB: change in systolic BP from baseline, Δ DBP: change in diastolic BP from baseline, NA: not applicable *p < 0.001.

**Table 5.** BP Goal Attainment At Visit Three In Patients Who Received Monotherapy And Those Who Received Dual Or More Therapy

	*Monotherapy*	*Dual or more therapy*
Non-diabetics	(*n* = 186)	(*n* = 106)
(a) SBP < 140 mmHg and DBP < 90 mmHg	112 (60.2)	44 (41.5)
(b) SBP < 140 mmHg [including patients counted in (a) above]	126 (67.7)	53 (50.0)
(c) DBP < 90 mmHg [including patients counted in (a) above]	134 (72.0)	62 (58.5)
Diabetics	(*n* = 49)	(*n* = 21)
(a) SBP < 130 mmHg and DBP < 80 mmHg	13 (26.5)	3 (14.3)
(b) SBP < 130 mmHg [including patients counted in (a) above]	21 (42.9)	5 (23.8)
(c) DBP < 80 mmHg [including patients counted in (a) above]	18 (36.7)	6 (28.6)

SBP: systolic blood pressure, DBP: diastolic blood pressure.

Compliance with the fixed-dose combination of ramipril/HCTZ was 89.2% at week four and 92.6% at week eight of the study (Table 3). In all, six patients reported eight different episodes of adverse events, with facial oedema and dry cough being reported twice by two different patients at separate times. Urinary retention, gout, repeated epistaxis and generalised body weakness were all reported once by different patients (Table 6). Adverse events occurred only in patients on half-standard or standard-dose therapy.

**Table 6.** Summary Of Reported Adverse Events

*Adverse event*	*Number of events*	*Severity*
Events possibly caused by study drug		
• Angio-oedema	2	Mild/moderate
• Urinary retention	1	Moderate
• Gout	1	Severe
• Dry cough	2	Severe
• Repetitive epistaxis	1	Severe
Events unlikely to have been caused by study drug		
• Asthenia and pain	1	Severe

The multivariate logistic regression analysis showed that in the ASTRAL study, diabetes was the most significant factor independently associated with BP goal attainment. The likelihood of achieving BP control in diabetic patients was 4.94 times less than in non-diabetic patients (OR: 4.92; 95% CI: 2.57–9.64; *p* < 0.05). Other factors associated with the attainment of BP goal were age (OR: 0.98; 95% CI: 0.97–0.99; *p* < 0.05), DBP (OR: 0.98; 95% CI: 0.97–0.99; *p* < 0.05) and SBP (OR: 0.99; 95% CI: 0.96–0.99; *p* < 0.05). However, with the logistic regression performed separately for diabetics and non-diabetics, no statistically significant predictors were found for goal achievement for diabetic patients. SBP at visit one was the only significant predictor of goal achievement in non-diabetic patients (OR: 0.969; 95% CI: 0.953–0.986; *p* = 0.0003).

## Discussion

High blood pressure is a major risk factor for cardiovascular morbidity and mortality worldwide. In sub-Saharan Africa, hypertension is one of the greatest health challenges after HIV/AIDS.^1^,^14^ Some key issues related to hypertension management in black Africans have been highlighted from this study: the high prevalence of hypertension-related co-morbidities; the high level of uncontrolled BP; the high prevalence of overweight and obesity among black Africans; the effectiveness of a combination of an ACE inhibitor and diuretic in controlling BP in Africans; the lower BP control achieved with an ACE inhibitor and diuretic combination in black African hypertensive patients with diabetes within the first eight weeks; and the good tolerability of the ACE inhibitor ramipril in black Africans.

Although there is evidence to suggest that ACE inhibitors lower BP to a lesser extent when used as monotherapy in African-Americans,^15^,^16^ the ASTRAL study has demonstrated that the combination of ACE inhibitor and thiazide diuretic was effective in controlling BP in black Africans. This is given that a high number of the patients reached target goals of BP control despite a substantial number of them either initially not receiving pharmacological treatment (8.9%) or being on a single agent for BP control (61.9%). The lower attainment of BP goals in the diabetic group, as was also indicated by the logistic regression analysis, may be an indication of the lower target and the degree of diabetic end-organ damage, including nephropathy.

Similar results were reported from South Africa where enalapril was ineffective as monotherapy but the addition of either a diuretic or reserpine dramatically increased BP control rates.^17^ Underscoring the efficacy of fixed-dose ramipril/HCTZ in this study was the sustained and significant change in SBP and DBP from week zero to week eight and the fact that most of the patients remained on standard or half-standard dose of ramipril/HCTZ throughout the study duration.

In addition to its effects on BP, ramipril offers additional benefits on target-organ protection in patients of African descent. In the African-American Study of Kidney Disease (AASK),^18^ ramipril was superior to amlodipine on renal outcomes in patients with hypertensive nephrosclerosis for comparable BP control. Other studies like HOPE^19^ and AIRE^20^ (although not directly linked to African patients because of small numbers of black patients enrolled), showed important benefits of ramipril in patients at high CV risk or with cardiac failure, respectively. The present study was however not designed to assess other additional benefits of ramipril.

During the ASTRAL study period, there were eight episodes of reported adverse events by six patients who were on either half-standard or standard therapy, suggesting that the adverse events may not have been dose related. It is nonetheless not surprising that facial oedema and dry cough were the two most common, given that these two, together with hyperkalaemia, hypotension and renal dysfunction, are known and commonly reported adverse effects of ACE inhibitor use.

In the HOPE study,^19^ reasons for discontinuation of the study were cough (7.3%), hypotension (1.9%) and angio-oedema (0.4%). However, given the short duration of the present study, it may be difficult to conclude that the study drug is safe with a small adverse-events profile, even though there has been a relatively low frequency of reported adverse events. A particular concern for clinicians in Africa may be the increased risk of angio-oedema^21^ and deaths^22^ reported with enalapril in African-Americans compared to whites, suggesting that there may be racial differences in the predilection to angio-oedema with the use of ACE inhibitors. In the ASTRAL study, the incidence of ACE inhibitor-related angio-oedema was 0.45%, which closely approximates the incidence reported in the HOPE study.^19^

One limitation of the ASTRAL study has been the inability to measure or document changes in serum electrolytes and certain metabolic parameters, such as sodium, potassium, uric acid and glucose, which are known to be affected by thiazide diuretics. The modern tendency is for low-dose HCTZ (12.5 mg) to be recommended to avoid these metabolic complications and this has been incorporated into recent guidelines.^23^ However HCTZ 12.5 mg as monotherapy has never been shown to improve outcomes and has a very weak anti-hypertensive activity in African patients.^16^ The higher dose of HCTZ (25 mg) offers more effective antihypertensive activity, particularly in combination with inhibitors of the renin–angiotensin system.

There are also concerns, particularly regarding the increased incidence of new-onset diabetes with the higher dose. This is a controversial topic but it has never been shown in a large outcome study that new-onset diabetes worsens outcomes.^24^ Although there was no measurement of blood glucose in this study there were no reported cases of new-onset diabetes.

A few patients were titrated upwards to ramipril 10 mg/HCTZ 50 mg for better BP control and although no significant adverse effects were reported, this raises some concerns about evaluating drug efficacy against its potential adverse effects, particularly electrolyte imbalances. Although this high dose of HCTZ has been used in major outcome studies, we would rather recommend the addition of alternative anti-hypertensive agents to achieve BP control until further safety information is available. In this study, the majority of patients did not require more than 25 mg HCTZ in the fixed-dose combination to achieve BP control.

## Conclusion

Fixed-dose combination of ramipril/HCTZ is an effective, tolerable anti-hypertensive agent with a good safety profile, which can be used to control BP in black Africans. This combination may be more effective in non-diabetics than patients with diabetes mellitus. Extended study with this combination is still needed to assess its long-term efficacy and safety.
